# Omentectomy with Gastrectomy for Gastric Cancer — Dilemma or Disease Demand? The STOMEC Study

**DOI:** 10.1007/s13193-025-02315-7

**Published:** 2025-04-29

**Authors:** Shivaji Sharma, Harish Kumar Hanumappa, Ravi Arjunan, Siddharth Jain, Syed Althaf, Chunduri Srinivas, Ali Zaid Anwar

**Affiliations:** 1 Department of Surgical Oncology, Dr. B. Borooah Cancer Institute, A. K. Azad Road, Guwahati, 781 016 India; 2 Department of Surgical Oncology, Kidwai Memorial Institute of Oncology, Bangalore, India; 3 Department of Surgical Oncology, Jawaharlal Nehru Cancer Hospital and Research Centre, Bhopal, India; 4 Department of Surgical Oncology, Dhanbad Medical College, Dhanbad, India

**Keywords:** Gastric cancer, Omentectomy, Omental resection, Omental preservation, Signet ring cell, Lymphovascular invasion

## Abstract

The omentum plays a valuable protective role post abdominal surgeries. Our goal was to determine the presence of metastatic omental lymph nodes or tumour deposits in stage I–III gastric adenocarcinoma, thus defining patterns of factors predictive of greater omental disease, which may contribute to omental sparing. Specimens of 115 patients with gastric adenocarcinoma stages I – III, operated consecutively, were analysed. The greater omentum was sent after separating it at 3 cm from the gastro – epiploic arcade. The number of total retrieved and metastatic lymph nodes in the greater omentum and the presence of tumour deposits, signet ring cell histology, and lympho-vascular invasion (LVI) of the primary tumour were noted. All calculations were carried out with IBM SPSS Statistics version 21.0. The Fisher exact chi-square test was done to evaluate the significance of association between the data, and a p-value ≤ 0.05 was considered to be significant. In total, 17.4% of patients had signet ring cell histology. A total of 34.8% were at pathological stage III. LVI was present in 25.2%. Two out of 115 patients (1.7%) had metastatic greater omental nodes, both of which were at stage III, signet ring cell histology, and LVI +ve. There was a significant association between malignant omental nodes, stage III disease, and signet ring cell type, while the association with LVI neared statistical significance. Irrespective of stage, the presence of omental disease in gastric adenocarcinoma is rare and may have a pattern of predictive features in its histology and stage, and thus set the stage for omental-sparing surgery in gastric cancer.

## Introduction

The stomach acts as the first storehouse of food after swallowing and passage through the oesophagus, and retains the food for 4–6 h before passing it distally. Gastric cancer is a common malignancy, with adenocarcinoma being the most common histopathologic type [1]. GLOBOCAN data reveal that 1,089,103 were newly diagnosed in 2020. The trend of it being predominantly prevalent in the South-east Asian countries is shifting, with an increasing incidence in the Middle Eastern region and parts of the erstwhile USSR [2].

The current standard of care for carcinoma of the stomach is a multi-modality approach which incorporates a surgical component in the form of resection of the tumour with adequate margins and D2 or modified D2 lymphadenectomy [3] along with chemotherapy ideally administered perioperatively due to its proven survival benefit [4]. However, the surgical component of gastric cancer management has been fraught with controversies— lymphadenectomy often finds itself in this spotlight; however, another aspect often swept under the carpet is a routine need for total omentectomy.

The omentum (derived from ancient Egyptians) [5] is a curtain-shaped structure which suspends itself from the greater curvature of the stomach and goes back up to attach to the transverse colon. It has aptly been termed the “policeman of the abdomen” by Rutherford Morison in 1906 [6], inasmuch that it goes about restoring order to any part of the peritoneal cavity that undergoes a disruption from the original milieu. The omentum has aggregates of macrophages, leukocytes, and immune cells — the so-called milky spots — which serve as a proliferation centre for immune cells [7, 8]. On one hand, omental milky spots are considered to be almost exclusive hot spots for localisation of disseminated tumour cells in the greater omentum [9], while on the other hand, it has a protective function in the post-operative period in preventing anastomotic leak, abdominal abscess, bleeding, and bowel ileus or adhesions [10].

The presence of omental metastasis indicates intra-peritoneal dissemination of disease, and the extent of its resection in cases where it is not involved is not well defined. The Japanese guidelines advocate total omentectomy for T3/T4 disease, but preservation of the omentum with a 3-cm margin along the gastro-epiploic (GEA) arcade for T1/T2 disease [3]; however, other guidelines offer no such specific recommendations. Thus, in light of the omentum having a significant post-operative protective action, and the evidence of omental hot spots being the first step for peritoneal dissemination, the need for routine total omentectomy is being questioned.

Ours is a prospective single-arm cohort study (Stomach cancer and the need for Omentectomy (STOMEC)) examining the presence of greater omental lymph nodes or metastatic deposits and their correlation with tumour stage and histopathologic features for patients undergoing curative surgical resection for adenocarcinoma of the stomach. The study was approved by the medical Ethical Committee.

## Materials and Methods

### Patient Selection

In total, 115 consecutive patients with gastric carcinoma who underwent sub-total or total gastrectomy from September 2021 to November 2022 at Kidwai Memorial Institute of Oncology (KMIO), Bengaluru, were selected for the study. Contrast-enhanced CT scans of the thorax, abdomen, and pelvis, as well as upper GI endoscopy with biopsy, were done preoperatively for all patients for staging and confirmation of diagnosis. The inclusion criteria were T1– T4/N0–N3/M0 patients with gastric adenocarcinoma who underwent a curative resection. Both peri-operative chemotherapy and upfront surgery groups were included. The exclusion criteria were histology, except adenocarcinoma, incompletely resected tumours, and pre-operative or intra-operative evidence of metastatic disease, including gross omental or peritoneal deposits or malignant ascites.

### Surgical Methods

#### Intra-Operative Steps

Upon opening the peritoneal cavity, any free fluid was sent for cytological analysis. Separate peritoneal wash for examination of cytology was not performed due to wide variance in reported positive cytology [11] and literature reporting negative cytology even in the presence of gross peritoneal disease [12]. Staging laparoscopy was avoided as the study coincided with the COVID-19 timeline to prevent aerosol borne infection as per institute protocol. The entire peritoneal cavity was inspected systemically for any grossly apparent disease. The greater omentum was divided from the hepatic to the splenic flexure of the transverse colon with monopolar cautery or LigaSure™ (Medtronic, Dublin, Ireland). Total omentectomy was done in all cases. All patients underwent a D2 or modified D2 lymphadenectomy.

#### Post-Operative Steps

**Fig. 1:**
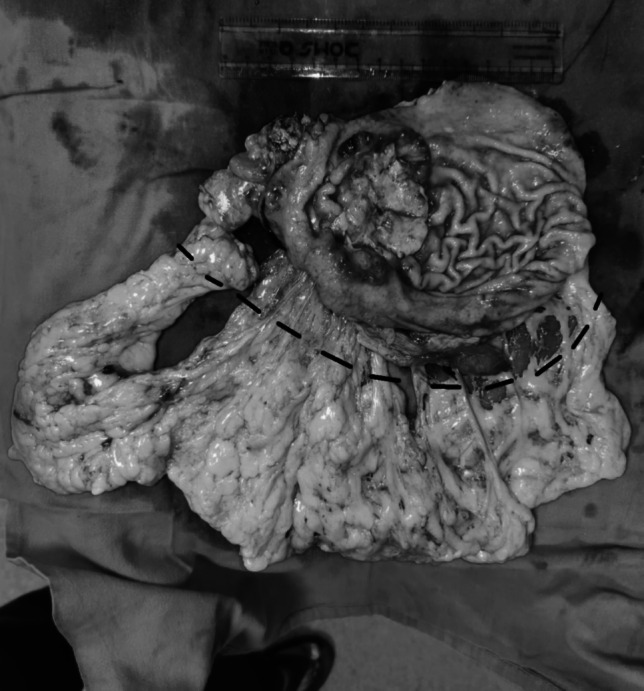
The resected specimen with omentum — the black dotted line marks the line of omental division

Once the specimen was removed, the greater curvature was marked along its entire length at a distance of 3 cm from the GEA, divided along this line, and sent separately for histopathology (Fig. 1). This 3-cm margin along the GEA was taken to leave an adequate margin for the station 2, 4, and 6 lymph nodes.

#### Pathologic Analysis

In the histo-pathology report, in addition to the assessment of the primary tumour and lymph nodes to classify as per pTNM, the greater omentum was assessed by making serial sections at 1-cm intervals and microscopic examination of these sections. The total number of lymph nodes or tumour deposits present in the greater omentum, the number of these lymph nodes that are positive for metastatic disease, and the presence of tumour deposits and lympho-vascular invasion (LVI) of the primary tumour were noted after haematoxylin and eosin staining.

## Statistical Analysis

Prior to the study, a minimum sample size of 110 patients was calculated using Cochrane’s formula to adequately power the study. All calculations were carried out with IBM SPSS Statistics version 21.0. The Fisher exact chi-square test was done to evaluate the significance of association between the data, and a *p*-value ≤ 0.05 was considered to be significant.

## Results

### Patient and Operative Details

A total of 115 patients consecutively operated with curative intent for carcinoma of the stomach in Kidwai Memorial Institute of Oncology were included in the study (Table 1). Of these, 65 were male (56.5%) and 50 were female (43.5%), with a median age of 55.4 years (range 30–90 years). In total, 90.4% of the patients had an ECOG performance status of 1 (*n* = 104) and 9.6% had an ECOG score of 2 (*n* = 11). The site of the malignancy was located in the proximal stomach (proximal to the incisura angularis, including the cardia, fundus, and body) in 22 patients (19.1%), who underwent a total radical gastrectomy, and distal in location in 93 (80.9%) patients (antro-pyloric — distal to the incisura angularis), and they underwent distal radical gastrectomy. Of these, 21 (18.3%) were diagnosed pre-operatively as ≥ T3/N+ and received peri-operative chemotherapy, while 94 (81.7%) received upfront surgery. The extent of lymphadenectomy was D1+ in 50 (43.5%) cases and D2 in 65 (56.5%).

**Table 1:** Patient and disease characteristics

Features	No. of patients (*n* = 115)
Gender	Male, *n *= 65 (56.5%)
	Female, *n *= 50 (43.5%)
Age	Mean, 55.4 yrs (range 30–90 yrs)
ECOG performance status	1, *n *= 104 (90.4%)
	2, *n *= 11 (9.6%)
Past history of malignancy	Yes, *n *= 1 (colon cancer)
	No, *n *= 114
Site of the gastric malignancy	Proximal, *n *= 22 (19.1%)
	Distal, *n *= 93 (80.9%)
Peri-operative chemotherapy	Yes, *n *= 21 (18.3%)
	No, *n *= 94 (81.7%)
Procedure performed	Distal gastrectomy, *n *= 93 (81.7%)
	Total gastrectomy, *n *= 22 (19.1%)
Extent of lymphadenectomy	D1+, *n *= 50 (43.5%)
	D2, *n *= 65 (56.5%)
Histopathology	Grade 1, *n *= 24 (20.9%)
	Grade 2, *n *= 35 (30.4%)
	Grade 3, *n *= 36 (31.3%)
	Signet ring, *n *= 20 (17.4%)
Pathological staging	Ia, *n *= 2 (1.7%)
	Ib, *n *= 18 (15.6%)
	IIa, *n *= 29 (25.2%)
	IIb, *n *= 24 (20.9%)
	IIIa, *n *= 16 (13.9%)
	IIIb, *n *= 13 (11.3%)
	IIIc, *n *= 11 (9.6%)
	pCR, *n *= 2 (1.7%)
Stage-wise distribution of signet ring cell histology	IIa, *n *= 1 (5%)
	IIb, *n *= 10 (50%)
	IIIa, *n *= 1 (5%)
	IIIb, *n *= 3 (15%)
	IIIc, *n *= 5 (25%)
Number of lymph nodes harvested	Mean 20.3 (range 5–56)
Number of lymph nodes positive for malignancy	Mean 2.7 (range 0–24)
Number of lymph nodes harvested from greater omentum alone	0.82 (range 0–20)
Presence of greater omental tumour deposits	None
LVI	Yes, *n *= 29(25.2%)
	No, *n *= 86 (74.8%)

### Pathological Assessment

In the final histopathology report, Grades 1, 2, and 3, and signet ring cell cancer (SRCC) were seen in 24 (20.9%), 35 (30.4%), 36 (31.3%), and 20 (17.4%) patients, respectively. The pathological staging was Ia, 2 (1.7%); Ib, 18 (15.6%); IIa, 29 (25.2%); IIb, 24 (20.9%); IIIa, 16 (13.9%); IIIb, 13 (11.3%); IIIc, 11 (9.6%); and pCR, 2 (1.7%). We also assessed the stage distribution of patients who had SRCC and found it predominantly in stages IIb (*n* = 10 (50% of all SRCC)) and III (*n* = 9 (45%)) — no SRCC was detected in stage I. The assessment of lymph nodes plays a significant aspect — a median of 19 lymph nodes was harvested overall for each patient (range 5–56), and a median of 2 lymph nodes (range 0–24) was positive for malignancy. From the greater omentum alone, 26 out of 115 patients had omental lymph nodes, with a median yield of 0.82 (range 0–20). Of these, only two patients had malignant nodes, and the rest were found to be reactive. LVI was present in 29 patients (25.2%). Both the patients with malignant omental nodes were stage III (T4aN2 and T4aN3b), SRCC, and had LVI (Table 2). No patient had malignant omental deposits.

**Table 2:** Details of the patients with positive omental nodes

Details	Patient 1	Patient 2
Stage	IIIb	IIIc
Proximal/distal	Distal	Proximal
NACT received	No	No
Lymph node dissection	D2	D2
Histology	Signet ring cell	Signet ring cell
Total no. of nodes harvested	21	23
Total no. of nodes +ve for malignancy	3	18
No. of omental nodes present	6	2
No. of omental nodes +ve for malignancy	1	1
LVI	Yes	Yes

### Results

We sought to find the statistical significance of the association between tumour stage, histology, and the presence or absence of malignant greater omental lymph nodes. Our statistical analysis (Table 3) revealed a significant association between the presence of greater omental metastatic lymph nodes and SRCC (*p* − 0.029), more so when the subgroup of stage III patients was considered (*p* − 0.044). The presence of LVI neared but failed to reach statistical significance (*p* − 0.062). Other factors, such as stages lower than III, location of the tumour, and grade 1–3 disease, had no significant association.

**Table 3:** Statistical analysis

	Total (*n *= 115)	No malignant omental nodes (*n *= 113)	Omental nodes positive for malignancy (*n *= 2)	***p***-value
Stage				
I	21 (18.4%)	21 (18.7%)	0 (0%)	0.667
II	52 (45.7%)	52 (46.5%)	0 (0%)	0.298
III	41 (35.9%)	39 (34.8%)	2 (100%)	0.125
Location				
Proximal	23 (20%)	22 (50%)	1 (50%)	0.361
Distal	92 (80%)	22 (50%)	1 (50%)	0.347
Histology				
Grade 1	22 (19.5%)	22 (19.8%)	0 (0%)	0.653
Grade 2	35 (30.9%)	35 (31.5%)	0 (0%)	0.507
Grade 3	36 (31.8%)	36 (32.5%)	0 (0%)	0.470
Signet ring	20 (17.7%)	18 (16.2%)	2 (100%)	**0.029**
LVI	29 (25.21)	27 (23.9)	2 (100%)	0.062
Stage III and signet ring	9 (7.8%)	7 (6.2%)	2 (100%)	**0.044**
LVI and signet ring	14 (12.1%)	12 (10.6)	2 (100%)	0.224
LVI and stage III	14 (12.1%)	12 (10.6%)	2 (100%)	0.192

## Discussion

The ancient Egyptians coined the word “omentum” from their practice to assess omens by looking at its pattern at the time of embalming bodies [13]. It is a sheath of loose mesothelial tissue comprising four layers and suspended from the greater curvature of the stomach, which extends down and then folds upon itself; thereafter, the fourth layer attaches to the transverse colon while the third layer proceeds up to line the lesser sac and anterior surface of the pancreas. The blood supply of the omentum is primarily from the GEA. As proposed by Griffith [14], the GEA gives off the right and left anterior omental arteries in the anterior layer of the omentum, and the right and left omental arteries in the posterior layer. These two groups of vessels anastomose with each other, giving rise to the “arc of Barkow” below the transverse colon. A few branches from the transverse and dorsal pancreatic arteries also contribute to the omental supply, making it richly vascularised.

“Milky spots” are aggregates of lymphocytes — predominantly macrophages — in the greater omentum that play a role in the removal of bacteria and other pathogens. It was initially seen that tumour macrophages have an anti-tumour effect against tumour cells in the omentum ex vivo; this is further amplified by the use of GM-CSF [15]. However, later studies revealed that tumour cells which gain access to the omentum preferentially grow in these milky spots, and the omental immune system is unable to get rid of the residual tumour cells; the tumour load in the omentum is greater in malignancies that have preferential peritoneal dissemination [9, 16]. Lymph nodes, however, were found to be absent from the greater omentum by Liebermann-Meffert et al. [17].

Thus, theoretically, the regional spread of gastric carcinoma, which occurs to the regional lymph nodes, should not occur in the omentum due to the relative absence of nodes. Meanwhile, the presence of tumour deposits in the greater omental milky spots qualifies as a metastatic disease. Although cytoreductive surgery (CRS) and hyperthermic intraperitoneal chemotherapy (HIPEC) are proven to be effective in gastric cancer [18], those studies included diseases which were pre-operatively identified by imaging (CT or MRI scans) and included complete resection of all metastatic disease followed by intra-peritoneal (IP) chemotherapy — this infers that only a total omentectomy, without a complete CRS and HIPEC, would not be sufficient in the event that the post-operative pathology reports the presence of disease in the greater omentum, as it implies the presence of peritoneal disease.

The omentum has several biological properties, including control of infection in the peritoneal cavity, tissue healing, neovascularisation, and prevention of post-operative adhesions and their sequelae, including bowel obstruction [10, 19]. This raises the query as to whether a routine omentectomy, irrespective of stage, histology, or systemic/IP therapy thereafter, has a role in reducing the tumour burden or predisposes the patient to debilitating post-operative morbidities.

The guidelines regarding the need and extent of omentectomy in gastric cancer are not well defined. The Japanese guidelines [3] recommend complete removal of the greater omentum (GO) for T3 or more tumours, while for lower T stages, the GO may be preserved beyond 3 cm from the GEA. Meanwhile, the ESMO guidelines make no specific recommendation regarding omental resection [20], while the NCCN recommends total removal of the GO along with a D1 lymphadenectomy at a minimum for resectable gastric cancers [21].

The unexplored and the undefined must be dealt with and clarified — “because it is there”. Hence, the dilemma of whether routine greater omentectomy accompanying radical surgery for gastric cancer has any survival benefit too has spurred a number of trials and research. Before assessing survival, prudence would dictate that we identify the risk factors that might predict a greater possibility of the presence of omental deposits. In a paper published by Haverkamp et al. [22] in 2016, the omentum was divided geographically into four quadrants to evaluate the distribution of lymph nodes and tumour deposits in relation to the location of the primary tumour. Out of a total of 50 patients, a metastatic omental lymph node was found in one patient, who was alive at a follow-up of 20 months. The tumour location was in the antrum of the stomach while the lymph node was in the right cranial quadrant proximal to the right gastro-epiploic artery. However, the distance from the GEA is not mentioned. Jongerius et al. in their OMEGA [23] trial sought to quantify the frequency of omental metastasis and the associated risk factors. In their study of 100 patients, 5% had omental deposits; all were stage III and had positive proximal or distal resection margins. Location was proximal stomach in three and linitis plastica in two out of these five; well or moderately differentiated in one and poorly differentiated or undifferentiated in four patients — the presence of SRCC was not mentioned. Barchi et al. [24] sought to corroborate this data further when they found in an analysis of 284 patients that a metastatic omental lymph node had a significant association with tumour size (mean size 8.06 cm for those with metastatic omental nodes), N stage (N2/N3), clinical stage (IIIb or IIIc), and venous invasion. This pointed the arrow towards possible identification of risk factors that might predict the presence of metastatic omental lymph nodes or deposits. Thus, total omentectomy could be restricted to patients with these risk factors identified pre- or intra-operatively while sparing the rest from the morbidity associated with the procedure at the cost of no additional harm.

Several studies tested this idea of selective omental preservation to assess the risks and benefits. S. Hasegawa et al. in 2012 [25] compared omentectomy vs. omental preservation for radical gastrectomy in gastric cancer — 3- and 5-year survival rates were 77.9% (range 69.5–86.3) and 66.6% (range 57.0–76.2) in the omental resection cohort and 89.3% (82.6–96.0) and 79.6% (68.2–91.0) in the omental preservation cohort — despite a difference in overall survival (OS), it did not reach statistical significance (*p* = 0.051); the difference in relapse-free survival (RFS) was not significant irrespective of T stage (T3 or T4) (*p* = 0.915). In patients with recurrence, the peritoneum was the most common site in both groups (7/98 in each) whether or not the omentum was preserved; this outcome questions the role of milky spots in the omentum as a sanctuary for residual/recurrent disease. Another meta-analysis published by Ho-Wei Lin et al. [26] in November 2021 compared survival outcomes for omentectomy vs. omental preservation (OP) for gastric malignancies; they found that pooled 5-year OS (RR 0.95, 95%CI 0.89–1.01) and DFS (RR 0.96, 95%CI 0.89–1.03) were in fact favourable for the OP group, although it did not reach statistical significance. The omentectomy group also had a slightly higher incidence of intra-peritoneal recurrence (RR 1.13, 95%CI 0.80–1.60, vs. RR 1.06, 95%CI 0.78–1.45) and higher intra-peritoneal complications (RR 1.15, 95%CI 0.89–1.50); again, significance was not reached. Also, the OP group had significantly shorter duration of surgery (MD 25.70, 95%CI 3.23–48.17) and lower blood loss (MD 56.29, 95%CI 14.02–98.56). Kim et al. [27] took it a step further and analysed survival data for OP in laparoscopic gastrectomy. Patients in whom intra-operatively serosa was seen to be involved (T4) underwent total omentectomy (TO) while serosal disease-free patients (≤ T3) underwent partial omentectomy (PO). They identified 19 recurrences in the follow-up period; 14/80 (17.3%) occurred in the TO cohort while 5/66 (7.6%) occurred in the PO group; this difference, although favouring the PO group, was not significant (*p* = 0.054), nor was DFS or DSS. In addition, PO was completed significantly faster and with less blood loss. Thus, even patients with T3 disease could undergo a PO safely. Meanwhile, the Japanese TOP-G trial [28] awaits its long-term results of overall and relapse-free survival for omental preservation vs. resection. Another Japanese trial — the JCOG1711, ROAD-GC [29] — seeks to demonstrate the non-inferiority of omental preservation in T3 or T4a gastric cancer — accrual is currently underway.

Hence, a number of studies seek to explore its safety in higher T stages. None of the studies, however, has found a correlation between the histology of the tumour and the risk of omental nodal spread or tumour deposits. Our study found that very few patients, even with locally advanced gastric cancer, had omental disease burden. Moreover, both patients with omental disease had SRCC, stage III disease, and the presence of LVI, of which stage III and SRCC were significantly associated with the presence of malignant omental nodes, while LVI neared but did not reach statistical significance. All these three factors can be determined pre- or intra-operatively via cross-sectional imaging, endoscopic ultrasound, or staging laparoscopy and can be a guide to TO or PO.

## Limitations

Our study had a few drawbacks. Firstly, peritoneal cytology was not taken into account. Secondly, the effect of peri-operative chemotherapy could not be assessed, as the number of patients with positive omental disease was not adequate for a sub-group analysis. Thirdly, due to the COVID-19 timeline, a staging laparoscopy was avoided, which possibly contributed to a stage migration of stage II or higher in 18.2% vs. 82.1% in the pre- vs. post-operative group, respectively. These questions, as well as the difference in survival and surgery-related morbidity, need to be carried out in a prospective study with a larger sample size and long-term follow-up, which will be our next step.

## Conclusion

Omental preservation can be extended to even T3 or T4 disease. However, selection on a case-to-case basis is of paramount importance, keeping in mind the histology and stage of the disease. A more discerning approach in selecting patients for omental preservation or resection may save patients from the morbidity associated with routine omental resection without affecting survival.

## Data Availability

The data supporting the findings of this study are available from the corresponding author upon reasonable request.
